# Bioprospecting and mode of action of volatile organic compounds from morphospecies of the endophytic fungus *Hypoxylon anthochroum* in the field of bioherbicides

**DOI:** 10.1007/s11274-025-04603-4

**Published:** 2025-10-13

**Authors:** Gonzalo Roque-Flores, Martha Lydia Macías-Rubalcava, Jacqueline Fuentes-Jaime, Celia Bustos-Brito, Rogerio Alejandro Saavedra-Barrera, Baldomero Esquivel

**Affiliations:** Departamento de Productos Naturales, Instituto de Química, Universidad Nacional Autónoma de México (UNAM), Ciudad Universitaria, Delegación Coyoacán, Ciudad de México, 04510 México

**Keywords:** *Hypoxylon anthochroum*, Endophytic fungus, Volatile organic compounds (VOCs), Phytotoxicity, Mitochondrial respiratory inhibitors, Membrane potential

## Abstract

In the present investigation, we explore the bioherbicidal activity of seven selected organic compounds (VOCs), previously identified as constituents of mixtures from four *Hypoxylon anthochroum* endophytic morphospecies. VOCs included four alcohols and three monoterpenes: 3-methyl-1-butanol, 2-methyl-1-butanol, 2-methyl-1-propanol and 2-phenylethanol, and eucalyptol, ocimene and terpinolene. Individual VOCs or VOCs’ mixtures exhibit strong bioherbicidal potential demonstrated through their phytotoxic effect on four herbaceous weed plants and one aquatic plant. VOCs demonstrated a significant inhibitory effect on the seed germination, root growth, and seedling respiration of four common herbaceous plants, including *Amaranthus hypochondriacus*, *Trifolium pratense*, *Medicago sativa* and *Panicum miliaceum.* In general, root growth was the most affected physiological process across all species, and *A. hypochondriacus* was the most affected. VOCs’ mixtures exhibit higher activity than pure compounds, at the same time, total mixture (*M*_*total*_; 7 VOCs) and the alcohol mixture (*M*_*alcohol*_; 4 VOCs) demonstrated greater bioherbicidal potential than terpene mixture (*M*_*terpene*_; 3 VOCs). In addition, we study the mode of action and mechanisms of phytotoxicity of VOCs evaluated on basal mitochondrial respiration and on the integrity or permeability of the mitochondrial membrane. In general, individual VOCs and VOCs’ mixtures have the potential to disrupt cellular respiration and mitochondrial membrane potential of intact mitochondria isolated from *M. sativa* seedlings. Once again, VOCs’ mixtures exhibit higher phytotoxicity on respiration and mitochondrial membrane potential than pure compounds, at the same time, *M*_*total*_ and *M*_*alcohol*_ present higher activity than *M*_*terpene*_. Finally, individual VOCs or VOCs’ mixtures showed phytotoxic effect on the aquatic plant *Lemna gibba*. Monoterpenes and VOCs’ mixtures proves to be the most effective. VOCs were proved to be promising candidates for the development of bioherbicides with specific targets of action.

**Graphical abstract:**

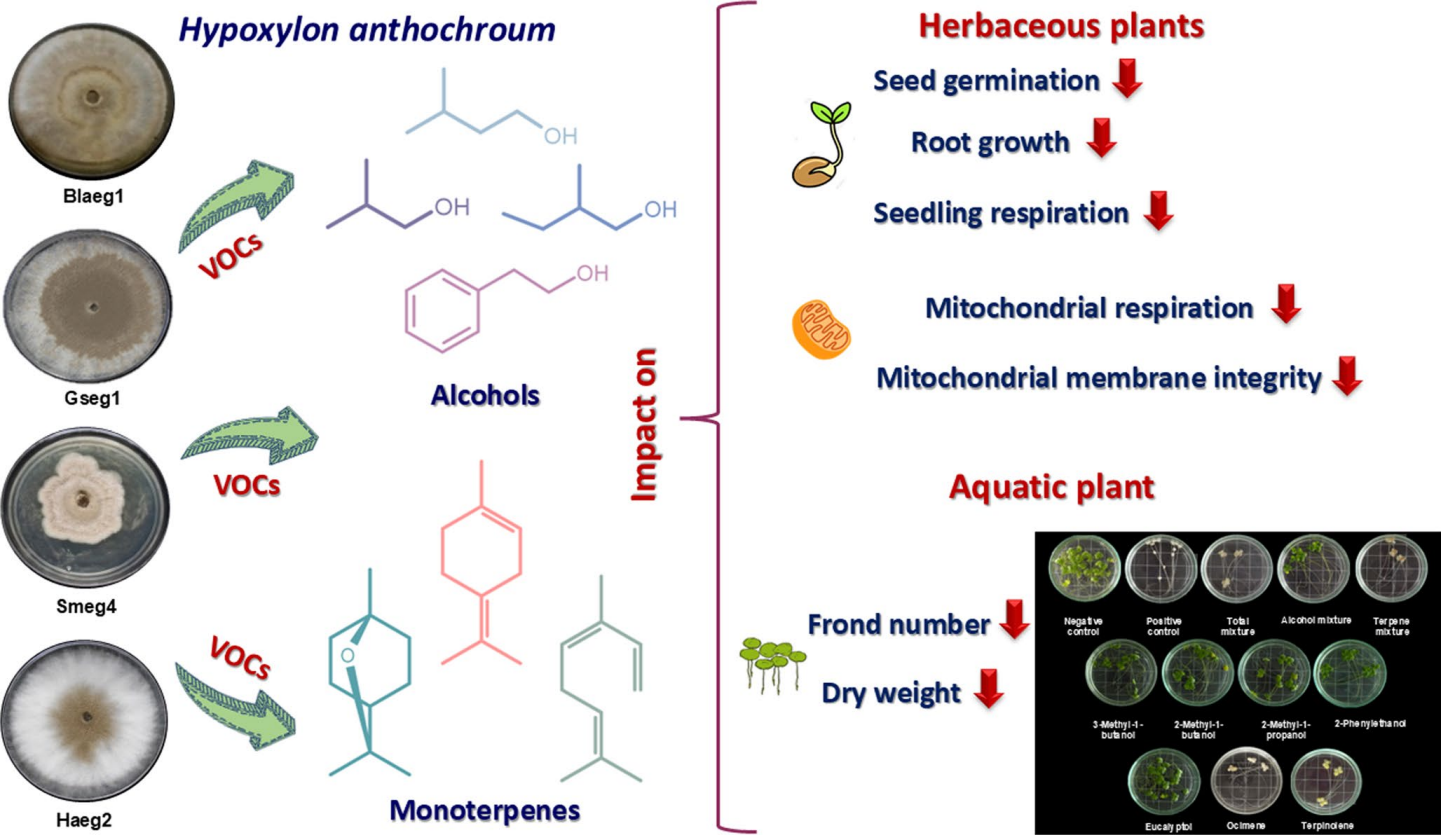

## Key points

• Endophytic fungus *Hypoxylon anthochroum* produces phytotoxic VOCs.

• VOCs inhibit basal oxygen consumption rate in isolated *M. sativa* mitochondria.

• Individual VOCs or VOCs’ mixtures altered the mitochondrial membrane potential.

## Introduction

From 2000 to 2022, the global production of primary crops experienced a substantial increase, reaching an estimated volume of 9.6 billion tons. This growth was primarily driven by significant advancements in sugarcane, corn, wheat, and rice production, which collectively accounted for nearly 50% of the total increase in production (FAO 2024). Concurrently, in 2022, the aggregate utilization of pesticides in the agricultural sector was estimated at 3.70 million tons, with herbicides being the most prominent group, accounting for approximately 2 million tons (FAO 2024). Herbicides have played a crucial role in the control of weeds, which are considered one of the main threats to agricultural crops due to the serious economic losses they can generate. In potato crops, for example, the presence of weeds caused an average yield loss of 44%, which represented an economic loss of US$465 million in the United States and US$61 million in Canada (Weed Science Society of America 2025). Although herbicides are widely used for the management of undesirable vegetation, their extensive and pervasive application has given rise to mounting concerns because of their deleterious effects on the environment, biodiversity, and food security. Moreover, this pattern of utilization has contributed to the emergence of herbicide-resistant weeds (Ayilara et al. 2023; Parven et al. 2025; Verdeguer et al. 2020). A global survey has documented 534 unique cases of resistant weeds, encompassing 21 of the 31 known sites of action and 168 distinct herbicides. Among the herbicides that have generated the largest number of resistant species are atrazine, glyphosate, and tribenuron-methyl (Heap 2025). In light of these challenges, there is an urgent need to explore sustainable alternatives, such as the utilization of bioherbicides, which are herbicides derived from natural sources. These bioherbicides not only effectively address the problem of weeds but also play a crucial role in mitigating the associated environmental risks (Macías-Rubalcava and Garrido-Santos 2022; Parven et al. 2025). In comparison with synthetic herbicides, bioherbicides exhibit superior selectivity, diminished environmental impact, and innovative mechanisms of action for utilization in biocontrol (Bendejacq-Seychelles et al. 2024; Schmaltz et al. 2023).

In this context, phytotoxic secondary metabolites of fungi have emerged as a promising source of bioherbicides (Bendejacq-Seychelles et al. 2024). A substantial body of research has been conducted on the genera *Alternaria*, *Fusarium*, *Phoma*, and *Trichoderma*, which have been identified as significant candidates for the study of bioherbicidal metabolites. A commercial bioherbicide derived from fungi is available for purchase under the brand name Bio-Phoma™. This bioherbicide is composed of *Phoma macrostoma*, while another commercial bioherbicide, Kichawi Kill™, is derived from *Fusarium oxysporum* f. sp. *strigae* (Ocán-Torres et al. 2024). Conversely, endophytic fungi capable of producing volatile organic compounds (VOCs) with potential applications in agriculture have received minimal attention (Macías-Rubalcava and Garrido-Santos 2022; Roy and Banerjee 2019). Among the endophytes with phytotoxic activity that were studied, ascomycetes from the genera *Muscodor*, *Xylaria*, and *Hypoxylon*, as well as basidiomycetes from the genus *Porostereum*, were found to be particularly notable. As demonstrated in the extant research, these have shown promise as biocontrol agents against weeds and/or as research tools for the development of more effective and less toxic alternative herbicides (Macías-Rubalcava and Garrido-Santos 2022).

Here, we explore the bioherbicide potential of seven selected VOCs, previously identified as constituents of VOCs’ mixtures from four *Hypoxylon anthochroum* endophytic morphospecies: Gseg1, Blaci, Haeg2, and Smeg4 (Macías-Rubalcava et al. 2018; Sánchez-Fernández et al. 2016; Ulloa-Benítez et al. 2016). The VOCs used included four alcohols and three monoterpenes belonging to two of the most abundant chemical groups found in bioactive VOCs’ mixtures produced by different species, including endophytic fungi of the genus *Hypoxylon* (Lee et al. 2016; Macías-Rubalcava et al. 2018; Muria-Gonzalez et al. 2020; Sánchez-Fernández et al. 2016; Sánchez-Ortiz et al. 2016; Sidorova et al. 2022; Suwannarach et al. 2013; Ulloa-Benítez et al. 2016). Furthermore, the majority of the selected VOCs exhibit a high degree of affinity for the SPME fiber, consequently being the most prevalent in the VOC mixtures produced by the four morphospecies. These compounds were identified through headspace solid-phase microextraction (HS-SPME) coupled to gas chromatography-mass spectrometry (GC-MS) (Macías-Rubalcava et al. 2018; Sánchez-Fernández et al. 2016; Ulloa-Benítez et al. 2016).

The phytotoxicity of these VOCs was assessed on the herbaceous plants *Medicago sativa*, *Trifolium pratense*, *Amaranthus hypochondriacus*, and *Panicum miliaceum*, as well as on the aquatic plant *Lemna gibba*, under both individual and combined treatments. To further investigate their mode of action, the effects of these VOCs on basal oxygen consumption and mitochondrial membrane permeability in *M. sativa* were also evaluated.

## Materials and methods

### Selected VOCs

**Fig. 1:**
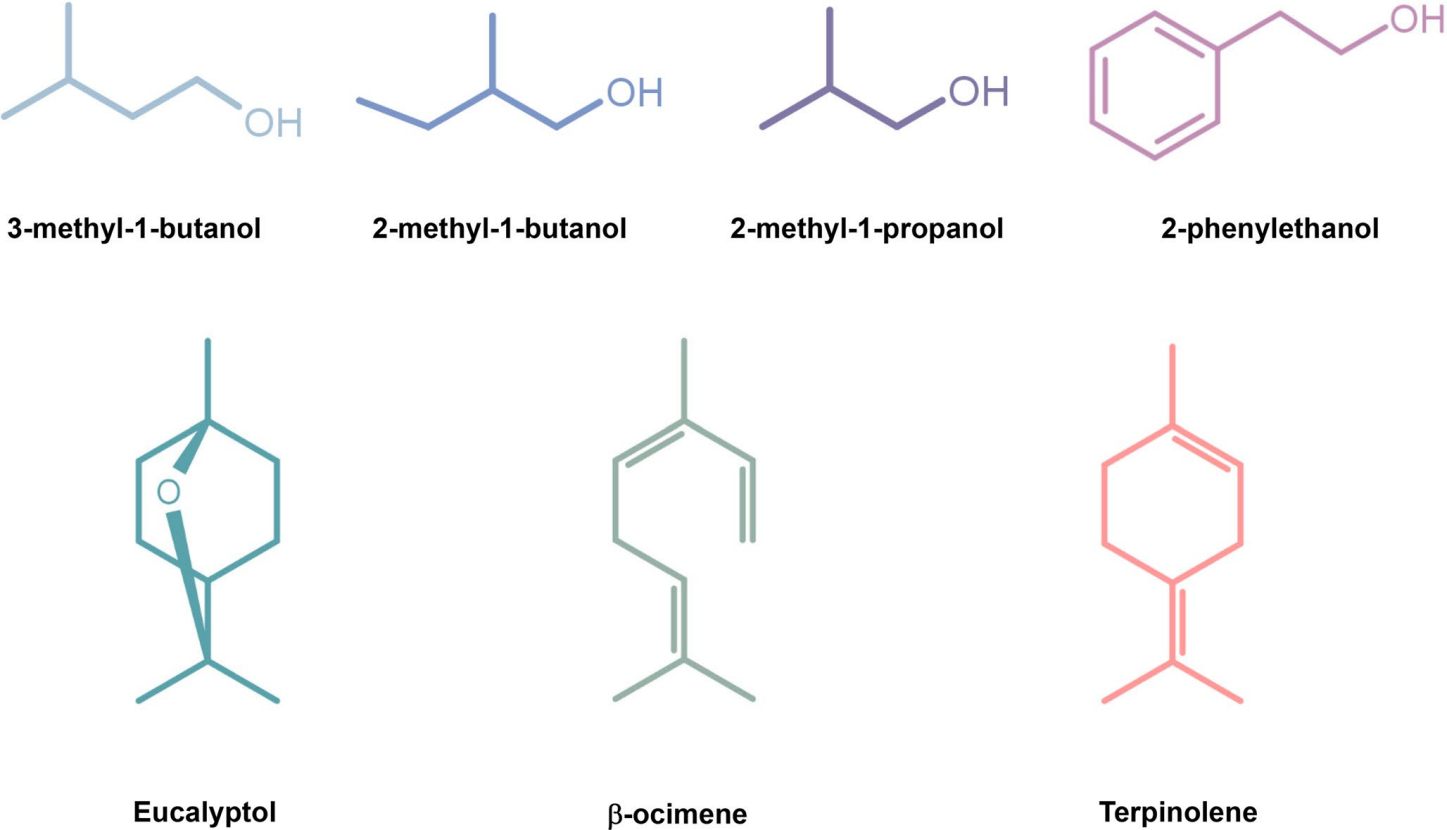
Selected volatile organic compounds (VOCs) produced by four *Hypoxylon anthochroum* endophytic morphospecies (Gseg1, Blaeg1, Haeg2, and Smeg4)

Seven VOCs, produced by four morphospecies of endophytic fungus *H. anthochroum*, which were previously identified as *H. anthochroum* morphospecies Gseg1 (*Nodulisporium* sp. GS4d2II1) (Macías-Rubalcava et al. 2018; Sánchez-Fernández et al. 2016), *H*. *anthochroum* morphospecies Blaci (Macías-Rubalcava et al. 2018; Ulloa-Benítez et al. 2016), *H*. *anthochroum* morphospecies Haeg2 and *H. anthochroum* morphospecies Smeg4 (Macías-Rubalcava et al. 2018) were selected: 3-methyl-1-butanol (isopentanol), (±)−2-methyl-1-butanol (sec-butyl carbinol), 2-methyl-1-propanol (isobutanol) and 2-phenylethanol (phenylethyl alcohol), and monoterpenes eucalyptol (1,8-cineole), (Z, E)- β-ocimene ((Z)−3,7-dimethyl-1,3,6-octatriene) and terpinolene (1-methyl-4-(1-methylethylidene) cyclohexene) (Fig. 1). Eucalyptol and 2-phenylethanol are biosynthesized by all four *H. anthochroum* endophytic morphospecies. Gseg1, Blaci, and Smeg4 were shown to biosynthesize 2-methyl-1-butanol, ocimene, and terpinolene. Gseg1 and Smeg4 were shown to biosynthesize 3-methyl-1-butanol, while Smeg4 were found to biosynthesize 2-methyl-1-propanol exclusively. All VOCs used in the bioassays were obtained from Sigma-Aldrich (St. Louis, Missouri, USA), with a purity ≥ 90%.

### Phytotoxic activity of VOCs from Hypoxylon anthochroum endophytic morphospecies on herbaceous weed plants

An evaluation was conducted of the *H. anthochroum* endophytic morphospecies regarding their impact on the germination, root growth, and seedling respiration (oxygen consumption rate) of three dicotyledonous plant species *A. hypochondriacus* (Amaranthaceae), *T. pratense* (Fabaceae), *M. sativa* (Fabaceae), and one monocotyledonous plant *P. miliaceum* (Poaceae). The seeds were obtained from Casa Cobo, S.A. de C.V., Central de Abastos, Mexico City, Mexico. These plants, commonly used in phytotoxic bioassays, were selected as test species due to their rapid, homogeneous, and high-frequency germination (Flores-Reséndiz et al. 2021; Ulloa-Benítez et al. 2016).

A total of seven selected VOCs were evaluated, including 3-methyl-1-butanol, 2-methyl-1-butanol, 2-methyl-1-propanol, 2-phenylethanol, and monoterpenes eucalyptol, ocimene, and terpinolene. These compounds were assessed using a gas phase method that was modified from Ulloa-Benítez et al. (2016). The following mixtures were tested: *M*_*total*_; 7 VOCs (total mixture); *M*_*alcohol*_; 4 VOCs (alcohol mixture); and *M*_*terpene*_; 3 VOCs (terpene mixture). The bioassays were performed in 5-cm-diameter Petri dishes containing 5 mL of 1% bacteriological agar. Just before the agar solidified, a small glass well (9- mm in diameter and 10- mm in height) was placed in the center of each Petri dish. Subsequently, 30 seeds were sown directly onto the agar, and immediately after, either one of the seven VOCs or a mixture of VOCs was added to the glass well. The negative control exhibited an empty glass well. Based on the initial bioherbicide potential evaluated at 1, 10, and/or 100 µg/mL, IC_50_ values were determined using a minimum of five distinct concentrations. The evaluation of the *M*_*total*_, *M*_*alcohol*_, and pure compounds (3-methyl-1-butanol, 2-methyl-1-butanol, and 2-methyl-1-propanol) was conducted within a concentration range of 0.5 µg/mL to 30 µg/mL. An evaluation of the concentration range between 50 and 1000 µg/mL was assessed on the following compounds: 2-phenylethanol, eucalyptol, ocimene, terpinolene and *M*_*terpene*_. The desired concentrations of the seven VOCs or VOCs’ mixtures were obtained by adding an aliquot of a chemical standard directly to a small glass well placed in the headspace of a 5-cm diameter Petri dish. The mixtures were prepared with each compound at the same concentration as indicated above. The Petri dishes were wrapped with four layers of Parafilm^®^ to prevent the loss of compounds and placed in a germination cabinet at 27 °C for 48 h under complete darkness (Thermo Scientific Precision, model 3759, Ohio, USA).

Subsequent to the incubation period, the quantity of non-germinated seeds was enumerated, the length of root growth was measured, and the oxygen consumption rates were quantified. It was contemplated that seed germination occurred when the testa was broken, with the subsequent emergence of the radicle and the seedlings having primary root sprouts > 1 mm. The measurement of respiration was achieved through the transfer of seedlings from each Petri dish to 10 mL glass chambers, with the addition of 3–5 mL of deionized water (depending on the root length) to each chamber. The amount of oxygen consumed by seedlings in the presence of individual VOCs or the mixtures was polarographically measured at 5-second intervals for a period of 3 min at a temperature of 27 °C. The measurement was taken using a Clark-type O_2_ electrode which was connected to a YSI^®^ model 5300 A oximeter (YSI Inc., Yellow Springs, OH). The results were expressed as percentages of inhibition, with the negative control set as 100% of respiration, and were determined through simple linear regression analysis of the generated curve (Flores-Reséndiz et al. 2021; Mead et al. 2002; Ulloa-Benítez et al. 2016).

### Effects of VOCs on basal oxygen consumption rate and on membrane permeability in isolated mitochondria

#### Isolation of mitochondria from Medicago sativa

The isolation of crude mitochondrial fractions was achieved by utilizing germinated *M. sativa* seeds and seedlings that were exposed to VOC mixtures and seven individual VOCs. *M. sativa* was selected as a test plant because its mitochondria exhibited the best performance, characterized by high membrane integrity, respiratory function, and favorable storage stability (Flores-Reséndiz et al. 2021). The gas phase method (see previous section) was employed in accordance with the methodologies described by Flores-Reséndiz et al. (2021), with a modification of the protocol. The following brief description outlines this modification.

From each Petri dish utilized in the phytogrowth-inhibitory bioassay, the 30 seeds and/or seedlings were collected and placed in a mortar. The isolation buffer, which contained 5 mM Tris-HCl, 300 mM sucrose, and 50 mM EDTA (pH 8.0), was added to the plant material in a 2:1 (w/v) ratio. The material was then subjected to vigorous maceration with the pestle for a period of three min. Notably, the procedure and all subsequent steps were carried out at 4 °C, with all materials pre-cooled and maintained on ice throughout the process. The cell homogenate was filtered through six layers of gauze, and the filtrate was subjected to centrifugation at 800 x*g* for 10 min. The pellet was then discarded, and the remaining fluid was subjected to centrifugation at 8,000 x*g* for 15 min. The mitochondrial pellet was resuspended in assay buffer containing 10 mM Tris-HCl, 250 mM sucrose, 150 mM KCl, 5 mM KH_2_PO_4_, and 1 mM MgCl_2_ (pH 7.4) until a concentration of 150 µg/mL was obtained. The resuspended pellet was then kept on ice until use. Protein concentration was determined by the Bradford method using bovine serum albumin as the standard (Bradford 1976). All reagents were procured from Sigma-Aldrich (St. Louis, Missouri, USA).

#### Effects of VOCs on mitochondrial basal respiration

The bioassay of the effects of VOCs’ mixtures and seven individual VOCs on basal oxygen consumption rate in isolated *M. sativa* mitochondria was carried out by placing 500–800 µL of crude mitochondrial fraction, corresponding to 150 µg/mL of total protein, into a 10 mL glass chamber. The assay buffer was added to achieve a final volume of 2 mL. Mitochondrial basal respiration was measured polarographically at 5-second intervals for approximately 5 min at 27 °C using a Clark-type O_2_ electrode connected to a YSI^®^ model 5300 A oximeter. TTFA (2-thenoyltrifluoroacetone) (Sigma-Aldrich, St. Louis, Missouri, USA), an inhibitor of the mitochondrial respiratory chain at the complex II level, ranging from 50 to 200 µg/mL, was utilized as the positive control. Crude mitochondrial extract obtained from *M. sativa* seeds germinated in the absence of treatment was utilized as a negative control. The mitochondrial oxygen consumption rate was determined by means of simple linear regression analysis of the generated curve. The results were expressed as percentages, with the negative control considered to be 100% of oxygen consumption (Flores-Reséndiz et al. 2021; Macías-Rubalcava et al. 2014; Mead et al. 2002).

#### Effects of VOCs on mitochondrial membrane integrity or permeability

To assess mitochondrial membrane potential (mΔψ) in intact *M. sativa* mitochondria following exposure to varying concentrations of VOCs’ mixtures and seven individual VOCs, fluorescence spectroscopy was employed, utilizing a TMRM dye (tetramethylrhodamine methyl ester) (Thermo Fisher Scientific, Waltham, MA, USA). The bioassay was conducted in 96-well flat-bottom microtiter plates. Crude mitochondrial extracts obtained from *M. sativa* seeds germinated in the absence of individual VOCs and VOCs’ mixtures and TTFA were used as negative and positive controls, respectively. A volume of 25 µL of crude mitochondrial fraction (150 µg/mL of protein) and 5 µL (20 µM) of TMRM fluorescent dye were added to each well. The final volume was adjusted to 200 µL with assay buffer. Subsequently, the flat-bottom well plates were subjected to an incubation period of 15 min at 27 °C, under conditions of darkness. Changes in mΔψ (membrane damage) at maximum TMRM fluorescence excitation and emission (λ_exc_ = 548 nm; λ_em_ = 574 nm) were recorded using a Cytation™ Model 5 spectrofluorometer (Winooski, VT, USA). The effect of individual VOCs and their mixtures on the integrity or permeability of the mitochondrial membrane was expressed as damage percentage and calculated according to the following equation (Flores-Reséndiz et al. 2021).

$${\%}\:\text{o}\text{f}\:\text{m}\text{e}\text{m}\text{b}\text{r}\text{a}\text{n}\text{e}\:\text{d}\text{a}\text{m}\text{a}\text{g}\text{e}=\:100-\left[\left(\frac{{RF}_{treatment}-{RF}_{blank}}{{RF}_{control}-{RF}_{blank}}\right)\ast100\right]$$

Where *RF* is the relative fluorescence.

## Effects of VOCs on the aquatic plant Lemna gibba

Plants were purchased at a local market in Xochimilco Mexico. The *L. gibba* stock culture was maintained in a climate incubator (Bioevopeak Co., Ltd., Shandong Province, China) at 23 ± 1 °C under 6600 lux of continuous cool white, fluorescent light. Cultures were maintained in 20X-AAP growth medium which contained 6 mM NaNO_3_, 1.18 mM MgCl_2_.6H_2_O, 0.61 mM CaCl_2_.2H_2_O, 1.18 mM MgSO_4_.7H_2_O, 0.13 mM K_2_HPO_4_.3H_2_O, 60 µM H_3_BO_3_, 42 µM MnCl_2_.4H_2_O, 12 µM FeCl_3_.6H_2_O, 16 µM Na_2_EDTA.2H_2_O, 0.48 µM ZnCl_2_, 0.12 µM CoCl_2_.6H_2_O, 0.60 µM Na_2_MoO_4_.2H_2_O, 1.4 nM CuCl_2_.2H_2_O, and NaHCO_3_ 1.19 mM (pH 7.4) in 1 L glass beakers with approximately 300 mL of medium. The total medium was renewed once a week. Tests with *L. gibba* were performed following the growth inhibition test described by Organization for Economic Co-operation and Development (OECD) (OECD-221 2006). A total of 12 fronds per pot (quadruplicates) were exposed for seven days under static conditions to a concentration of 300 µg/mL of each VOC, using glass Petri dishes with 10 mL of culture medium. To calculate growth inhibition, at the end of the experiment, the number of fronds was determined by direct count. Plants were then placed in an oven at 60 °C for 48 h to determine dry weight. The culture medium was used as the negative control, and Rival^®^ [glyphosate: N-(phosphonomethyl) glycine] (Monsanto, São Paulo, Brazil) at 100 µg/mL was used as the positive control. All reagents used in the culture media for *L. gibba*, according to the OECD (OECD 221 2006) guidelines, were purchased from Sigma-Aldrich, with a purity > 98%.

### Statistical analysis

The data from the various bioassays (germination, root growth, seedling respiration, mitochondrial basal respiration, changes in mΔψ, and *L. gibba*) were obtained on three separate occasions under a randomized design, with four replicates per treatment. These data were analyzed using analysis of variance (ANOVA) to determine significant differences from the control group. Tukey’s Honestly Significant Difference (HSD) test or Dunnett’s method was applied to establish significant differences between the various treatments. Additionally, an analysis was conducted to determine the IC_50_ value, defined as the concentration of VOCs that could inhibit the effect by 50%, using log-probit analysis based on percent inhibition data. Calculations were performed using GraphPad Prism version 10.4.2 (San Diego, CA, USA). The data are expressed as the mean ± standard deviation (SD), and values of *P* < 0.05 were considered statistically significant (Flores-Reséndiz et al. 2021; Mead et al. 2002; Zar 2010).

## Results

### Phytotoxic effects of VOCs on herbaceous weed plants

#### Total mixture of VOCs

**Fig. 2:**
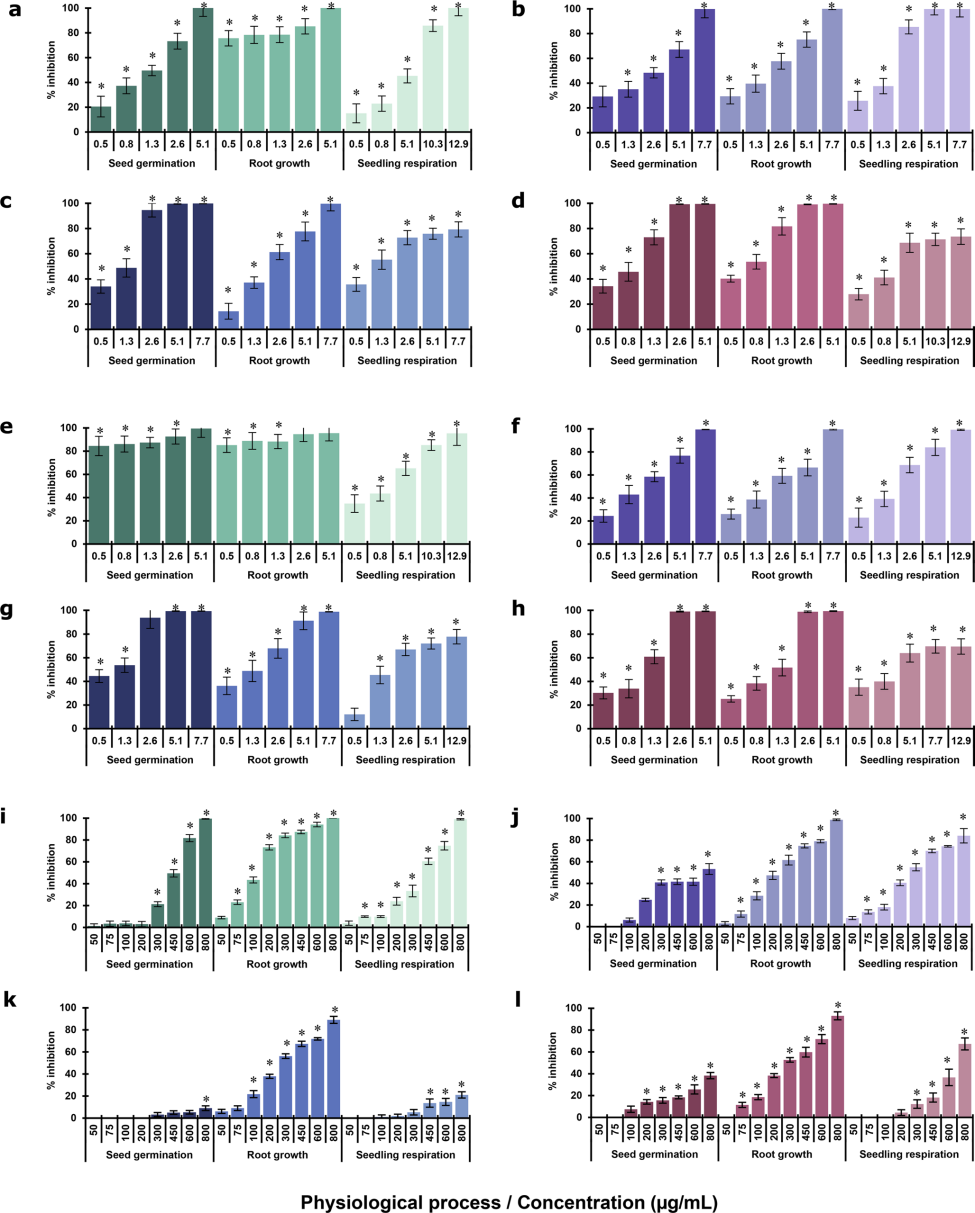
Phytotoxic effect of VOC mixtures or individual VOCs on seed germination, root growth and seedling respiration of *Amaranthus hypochondriacus* (green), *Panicum miliaceum* (purple). *Trifolium pratense* (blue) and *Medicago sativa* (pink). **a**-**d** Total mixture (*M*_*total*_;7 VOCs). **e**-**h** Alcohol mixture (*M*_*alcohol*_; 4 VOCs;). **i**-**l** Terpene mixture (*M*_*terpene*_; 3 VOCs). Vertical bars represent SD, 4n = 4; ANOVA * *P* < 0.05 compared with the negative control

As illustrated in Fig. 2a and d, evaluation with *M*_*total*_ showed a significant, concentration-dependent phytotoxic effect on all four test plants. The germination and root growth of *A. hypochondriacus* (Fig. 2a), *P. miliaceum* (Fig. 2b), *T. pratensis* (Fig. 2c), and *M. sativa* (Fig. 2d) were inhibited by more than 50% at 0.5–2.6 µg/mL. With the exception of *A*. *hypochondriacus*, which showed 40% inhibition, respiration in the other plants was reduced by more than 65% at 5.1 µg/mL. Overall, most physiological processes in the four plants were completely inhibited.

#### Alcohol mixture

In general, as shown in Fig. 2e and h, evaluation of *M*_*alcohol*_ revealed a significant, concentration-dependent phytotoxic effect on all test plants. At low concentrations of 0.5–2.6 µg/mL, the mixture inhibited germination and root growth by more than 90% in *A. hypochondriacus* (Fig. 2e), the most sensitive species. In *P. miliaceum* (Fig. 2f) and *T. pratensis* (Fig. 2g), all three physiological showed over 60% inhibition at 2.6 µg/mL, whereas the germination and root growth of *M. sativa* (Fig. 2h) were affected by over 50% at 1.25 µg/mL. Furthermore, respiration was inhibited by over 65% in all plants at 5.1 µg/mL. At higher concentrations (2.6–12.9 µg/mL), most physiological processes were inhibited by over 95% across the four plants, except for respiration in *T. pratensis* and *M. sativa.*

#### Monoterpenes mixture

In general, evaluation of *M*_*terpene*_ revealed a significant, concentration-dependent phytotoxic effect on the three physiological processes in all four plant species. Root growth was inhibited by more than 50% in all species at 300 µg/mL. Respiration of *A. hypochondriacus* (Fig. 2i) and *P. miliaceum* (Fig. 2j) was also reduced, showing more than 60% inhibition at 450 µg/mL. Among the species tested, *A. hypochondriacus* seeds were the most sensitive, with all three physiological processes reaching 100% inhibition at 800 µg/mL.

#### Alcohols evaluated individually

**Fig. 3:**
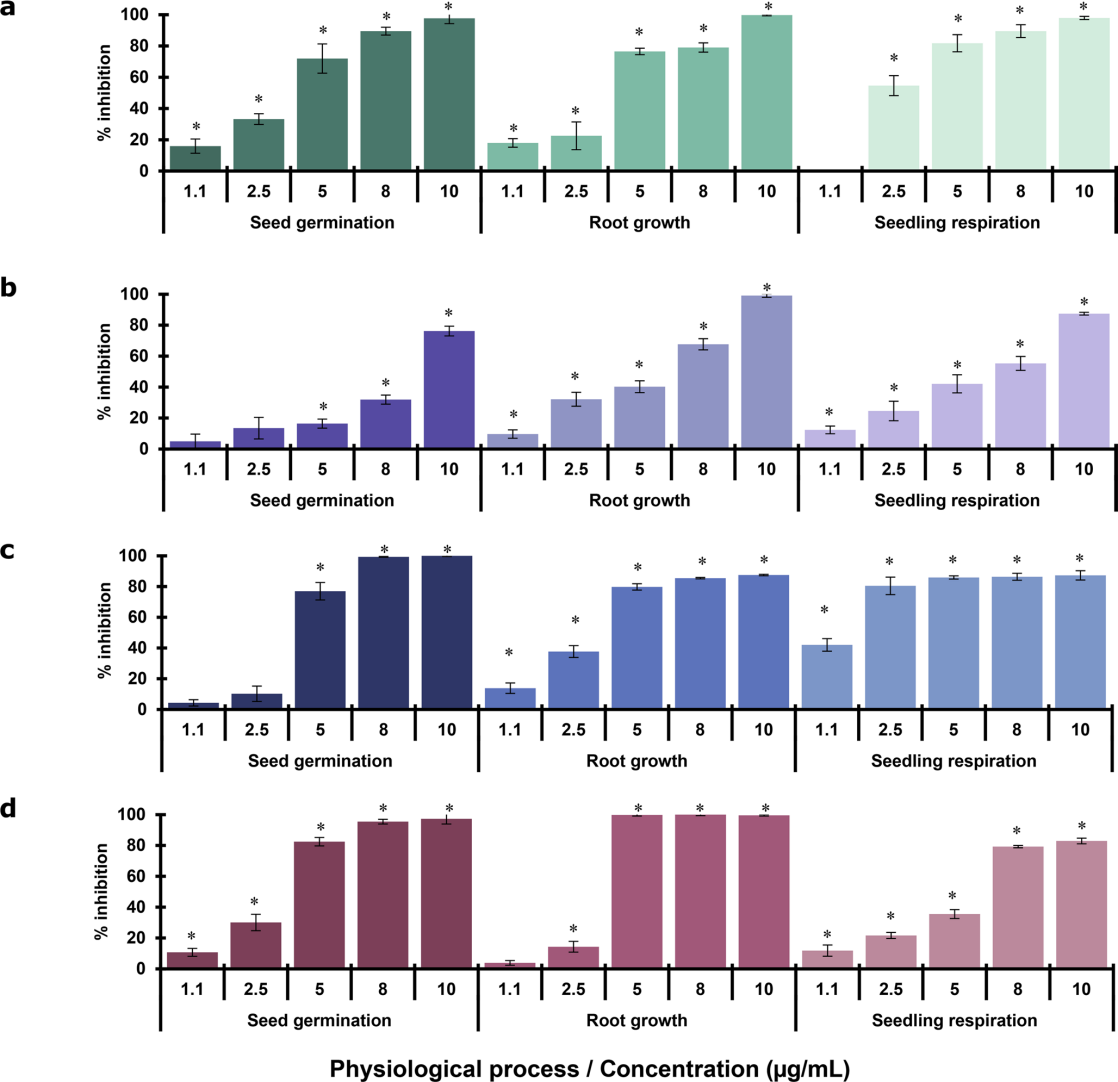
Phytotoxic effect of 3-methyl-1-butanol on seed germination, root growth and seedling respiration of *Amaranthus hypochondriacus* (green), *Panicum miliaceum* (purple), *Trifolium pratense* (blue) and *Medicago sativa* (pink). Vertical bars represent SD, 4n = 4; ANOVA * *P* < 0.05 compared with the negative control

In general, evaluation of the four individual alcohols demonstrated a significant, concentration-dependent inhibitory effect on the three physiological processes in all four test species. Among them, 3-methyl-1-butanol was the most potent, causing complete inhibition of *M. sativa* root growth at 5 µg/mL. Overall, this compound inhibited at least 80% of the processes at 10 µg/mL, except for *P. miliaceum* germination (76.21%). 2-methyl-1-butanol completely inhibited the processes in *A. hypochondriacus* and *T. pratensis* at 15 µg/mL, whereas *P. miliaceum* and *M. sativa*, inhibition ranged from 35% to 60% at equivalent concentrations (Fig. 3). 2-methyl-1-propanol inhibited more than 60% of the processes at 75 µg/mL, except for *T. pratensis* respiration. Overall, this compound showed an inhibition of more than 95% in the processes of *A. hypochondriacus* and *M. sativa*. Finally, 2-phenylethanol showed the strongest effect on root growth, producing at least 50% inhibition in all species at 125 µg/mL, with *A. hypochondriacus* being the most susceptible, showing 70–100% inhibition at 500 µg/mL (data not shown).

#### Monoterpenes evaluated individually

Among the monoterpenes tested, eucalyptol was the only compound that exhibited significant phytotoxic activity in all four test species, primarily affecting root growth. This process was the most impacted in all species, with 90% inhibition observed at the maximum concentration of 500 µg/mL. *A. hypochondriacus* seeds showed the greatest sensitivity, with 95% inhibition of germination and 72% inhibition of respiration at 500 µg/mL, along with more than a 50% reduction in root growth at 125 µg/mL (data not shown).

The IC_50_ values in Table 1 corroborate that, in general, root growth was the most affected physiological process, and that *A. hypochondriacus* seeds were the most sensitive. The most phytotoxic treatments were *M*_*total*_ and *M*_*alcohol*_, with IC_50_values ranging from less than 0.5 µg/mL to 1.8 µg/mL. In contrast, the *M*_*terpene*_ values ranged from 122 µg/mL to more than 800 µg/mL. Among the individual compounds, 3-methyl-1-butanol demonstrated the greatest phytotoxic potential, with IC_50_values ranging from less than 2.5 µg/mL to 8.2 µg/mL. Lastly, eucalyptol was the only monoterpene that significantly affected root growth, with IC_50_values between 98 µg/mL and 291 µg/mL.

**Table 1:** Phytogrowth-inhibitory activity. IC_50_ values of VOCs’ mixtures or individual VOCs from four *Hypoxylon anthochroum* endophytic morphospecies on seed germination, root elongation and seedling respiration of *Panicum miliaceum*, *Medicago sativa*, *Trifolium pratense* and *Amaranthus hypochondriacu*s

Treatment	Seed	IC_50_ µg/mL (mM)*		
		Germination	Root growth	Seedling Respiration
Seven VOCs mixture (*M*_*total*_)	*A. hypochondriacus*	1.1	< 0.5	1.7
	*P. miliaceum*	1.9	1.6	1.3
	*T. pratense*	0.9	1.8	0.9
	*M. sativa*	0.7	0.6	1.6
Alcohols mixture (*M*_*alcohol*_)	*A. hypochondriacus*	< 0.5	< 0.5	1.2
	*P. miliaceum*	1.6	1.7	1.5
	*T. pratense*	0.7	1.0	1.8
	*M. sativa*	0.9	0.9	1.6
Terpenes mixture (*M*_*terpene*_)	*A. hypochondriacus*	428	122	353
	*P. miliaceum*	653	210	257
	*T. pratense*	> 800	261	> 800
	*M. sativa*	> 800	278	667
2-phenylethanol	*A. hypochondriacus*	254 (2.079)	97 (0.794)	294 (2.407)
	*P. miliaceum*	> 500 (4.093)	174 (1.424)	> 500 (4.093)
	*T. pratense*	> 500 (4.093)	278 (2.276)	> 500 (4.093)
	*M. sativa*	> 500 (4.093)	291(2.382)	> 500 (4.093)
3-Methyl-1-butanol	*A. hypochondriacus*	3.1 (0.035)	3.5 (0.040)	< 2.5 (0.028)
	*P. miliaceum*	8.2 (0.093)	5.5 (0.062)	5.1 (0.058)
	*T. pratense*	3.8 (0.043)	2.9 (0.033)	1.3 (0.018)
	*M. sativa*	3.1 (0.034)	2.9 (0.033)	5.2 (0.059)
2-Methyl-1-butanol	*A. hypochondriacus*	10.2 (0.116)	10.6 (0.120)	10.2 (0.116)
	*P. miliaceum*	12.1 (0.137)	12.3 (0.140)	> 15 (0.170)
	*T. pratense*	6.9 (0.078)	6.2 (0.070)	8.4 (0.095)
	*M. sativa*	> 15 (0.170)	14.7 (0.167)	> 15 (0.170)
2-Methyl-1-propanol	*A. hypochondriacus*	35.5 (0.479)	33.4 (0.450)	41.3 (0.557)
	*P. miliaceum*	54.8 (0.739)	53.6 (0.723)	48.9 (0.659)
	*T. pratense*	69.7 (0.940)	36.7 (0.495)	> 75 (1.012)
	*M. sativa*	35.2 (0.475)	24.5 (0.330)	51.3 (0.692)
Eucalyptol	*A. hypochondriacus*	255 (1.653)	98 (0.635)	294 (1.906)
	*P. miliaceum*	> 500 (3.242)	174 (1.128)	> 500 (3.242)
	*T. pratense*	> 500 (3.242)	278 (1.802)	> 500 (3.242)
	*M. sativa*	> 500 (3.242)	291 (1.887)	> 500 (3.242)
Ocimene	*A. hypochondriacus*	> 500 (3.670)	> 500 (3.670)	> 500 (3.670)
	*P. miliaceum*	> 500 (3.670)	> 500 (3.670)	> 500 (3.670)
	*T. pratense*	> 500 (3.670)	> 500 (3.670)	> 500 (3.670)
	*M. sativa*	> 500 (3.670)	> 500 (3.670)	> 500 (3.670)
Terpinolene	*A. hypochondriacus*	> 500 (3.670)	> 500 (3.670)	> 500 (3.670)
	*P. miliaceum*	> 500 (3.670)	> 500 (3.670)	> 500 (3.670)
	*T. pratense*	> 500 (3.670)	> 500 (3.670)	> 500 (3.670)
	*M. sativa*	> 500 (3.670)	> 500 (3.670)	> 500 (3.670)

* The IC₅₀ values of individual VOCs were expressed in µg/mL and, in parentheses, in mM.

Data are the mean of three replicates under a random design with four repetitions per treatment.

### Effect of VOCs on respiration and mitochondrial membrane damage

In general, individual VOCs or VOCs’ mixtures had a significant, concentration-dependent effect on respiration and mitochondrial membrane damage in intact *M. sativa*.

#### Mixtures of VOCs

**Fig. 4:**
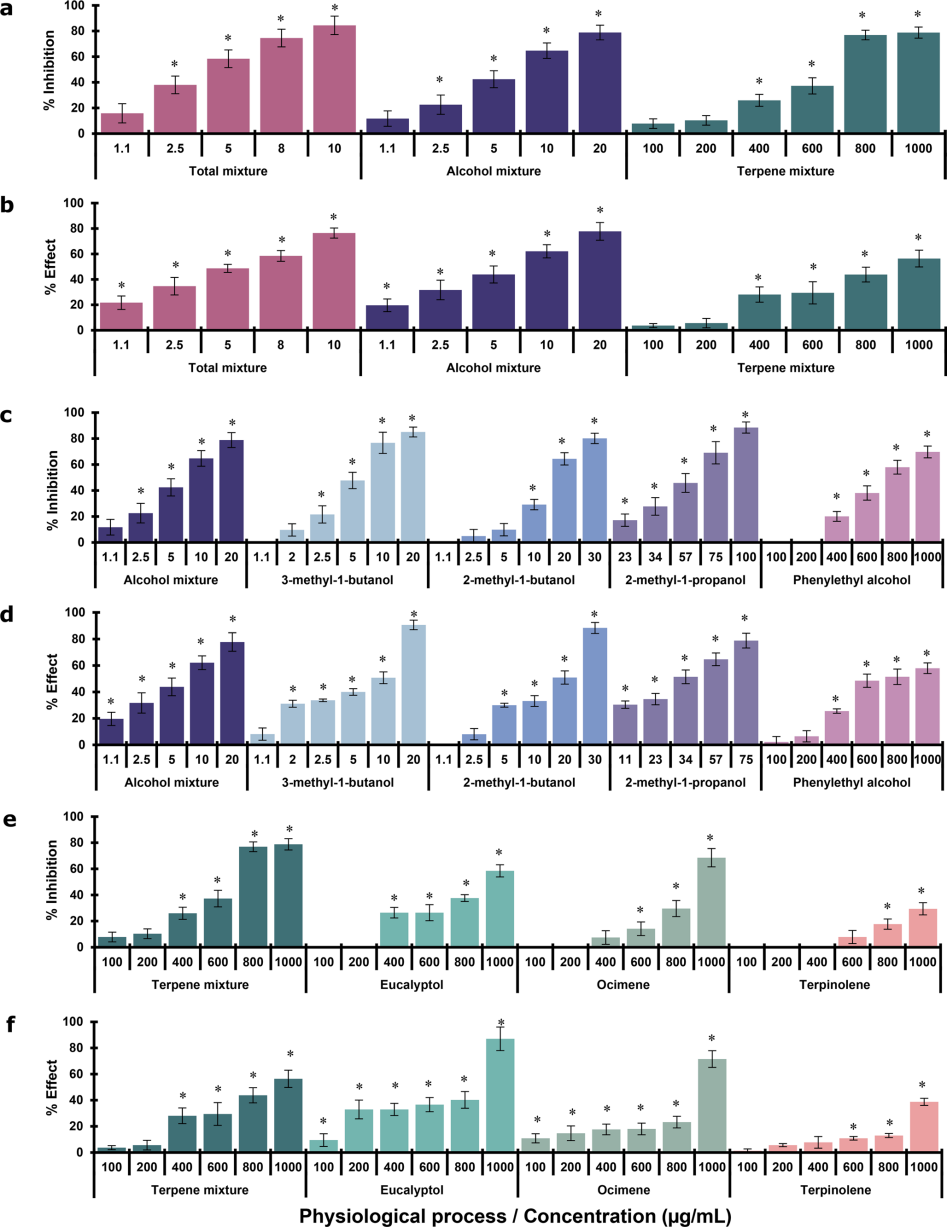
Phytotoxic effect of VOC mixtures or individual VOCs on basal oxygen consumption rate (**a**, **c** and **e**) and on membrane permeability (**b**, **d** and **f**) of isolated *Medicago sativa* mitochondria. Vertical bars represent SD, 4n=4; ANOVA * *P *< 0.05 compared with the negative control

Treatment with *M*_*total*_ and *M*_*alcohol*_ inhibited mitochondrial respiration by at least 40% at 5 µg/mL, reaching a maximum effect of approximately 80% at 20 µg/mL. In contrast, *M*_*terpene*_ achieved a similar 80% inhibition only at a significantly higher concentration of 800 µg/mL (Fig. 4a).

The *M*_*total*_ was the most potent in impairing mΔψ, causing at least 50% damage at 5 µg/mL and approximately 80% at 10 µg/mL. While *M*_*alcohol*_ also induced nearly 80% membrane damage, a higher concentration of 20 µg/mL was required. The *M*_*terpene*_ was the least potent, causing about 60% damage at 1000 µg/mL (Fig. 4b).

#### Alcohols evaluated individually

As illustrated in Fig. 4c and d, the individual alcohols exhibited a clear concentration-dependent inhibitory effect on mitochondrial respiration and mΔψ. 3-methyl-1-butanol and 2-methyl-1-butanol inhibited respiration by at least 60% at 20 µg/mL, reaching a maximum effect of about 80% at concentrations of 10 and 30 µg/mL, respectively. 2-methyl-1-propanol inhibited about 50% of respiration at 57 µg/mL, with a maximum effect of approximately 90% at 100 µg/mL. 2-phenylethanol was the least potent, causing only 40% inhibition at 600 µg/mL and a maximum of about 70% at 1000 µg/mL.

The mΔψ was affected by more than 50% by 3-methyl-1-butanol and 2-methyl-1-butanol at concentrations of 10 and 20 µg/mL, respectively. The maximum damage, of about 90%, was reached at 20 and 30 µg/mL, respectively. 2-methyl-1-propanol also compromised the membrane by more than 50% at 34 µg/mL, with a maximum damage of approximately 80% at 75 µg/mL. Finally, 2-phenylethanol was the least potent, causing about 50% damage at 600 µg/mL and a maximum of approximately 60% at 1000 µg/mL (Fig. 4d).

#### Monoterpenes evaluated individually

Eucalyptol and ocimene were the most potent inhibitors of mitochondrial respiration at 1000 µg/mL, with an inhibition of about 60% and 70% respectively, while terpinolene caused a lesser effect, about 30% (Fig. 4e).

The mΔψ was most affected by eucalyptol, which caused about 90% damage at 1000 µg/mL. Ocimene and terpinolene, on the other hand, caused less damage, about 70% and 40% respectively, at the same concentration (Fig. 4f).

The IC_50_ values of individual VOCs or VOCs’ mixtures on basal mitochondrial respiration and membrane damage showed that *M*_*total*_ and *M*_*alcohol*_ were the most phytotoxic treatments, with IC_50_ values below 6.5 µg/mL for both processes. In sharp contrast, the IC_50_ values for *M*_*terpene*_ were 627 µg/mL for respiration and 893 µg/mL for membrane damage (Table 2). The most potent individual alcohols were 3-methyl-1-butanol and 2-methyl-1-butanol, with IC_50_ values of 5.5 and 14.8 µg/mL for respiration and 5.9 and 15.2 µg/mL for membrane damage, respectively. 3-methyl-1-butanol was approximately five times more potent than the positive control TTFA, while 2-methyl-1-butanol was approximately two times more potent in both studied physiological processes.

**Table 2:** IC_50_ values of the VOCs’ mixtures or individual VOCs from four *Hypoxylon anthochroum* endophytic morphospecies on basal oxygen consumption rate, and on membrane permeability of isolated *Medicago sativa* mitochondria

Treatment	IC_50_ µg/mL (mM)*	
	Mitochondrial basal respiration	Mitochondrial membrane damage
Seven VOCs mixture^a^	4.6	6.3
Alcohols mixture^a^	5.7	5.8
Terpenes mixture^a^	627	893
Phenylethyl alcohol	706 (5.779)	783 (6.410)
3-Methyl-1-butanol	5.5 (0.062)	5.9 (0.067)
2-Methyl-1-butanol	14.8 (0.168)	15.2 (0.172)
2-Methyl-1-propanol	30.6 (0.413)	53.0 (0.715)
Eucalyptol	902 (5.848)	510 (3.306)
Ocimene	885 (6.496)	---
Terpinolene	> 1000 (> 0.734)	> 1000 (> 0.734)
TTFA^a^	71 (0.320)	79 (0.356)

* The IC₅₀ values of individual VOCs were expressed in µg/mL and, in parentheses, in mM.

^a^ Positive control TTFA (2-thenoyltrifluoroacetone).

---With the experimental values it was not possible to calculate the IC_50_.

Data are the mean of three replicates under a random design with four repetitions per treatment.

### Phytotoxic effects of VOCs on the aquatic plant Lemna gibba

**Fig. 5:**
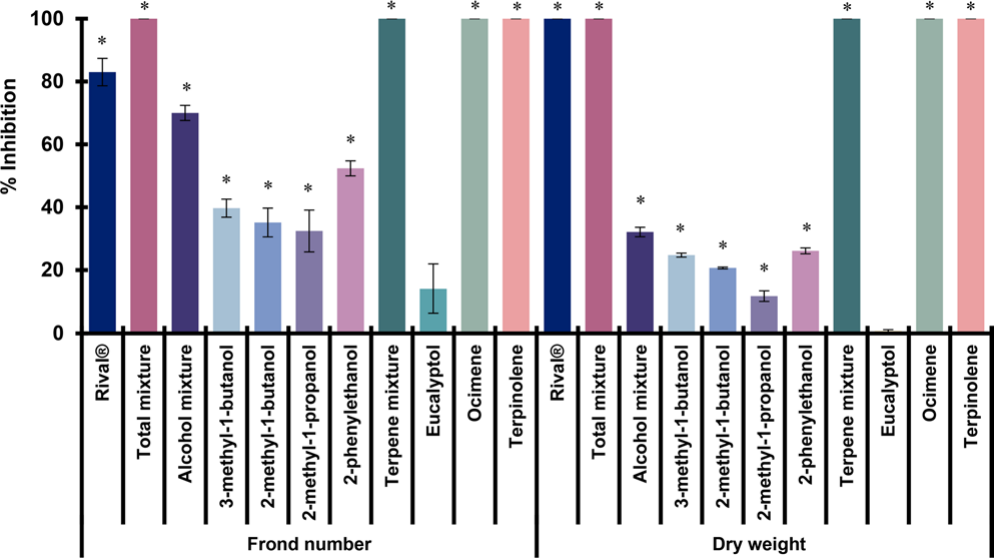
Phytotoxic effect of VOC mixtures or individual VOCs at 300 μg/mL on frond numbers and dry weights of the aquatic plant *Lemna gibba*. Rival^®^ [glyphosate: *N-*(phosphonomethyl)glycine] was used as positive control (300 μg/mL). Vertical bars represent SD, 4n=4; ANOVA * *P *< 0.05 compared with the negative control

**Fig. 6:**
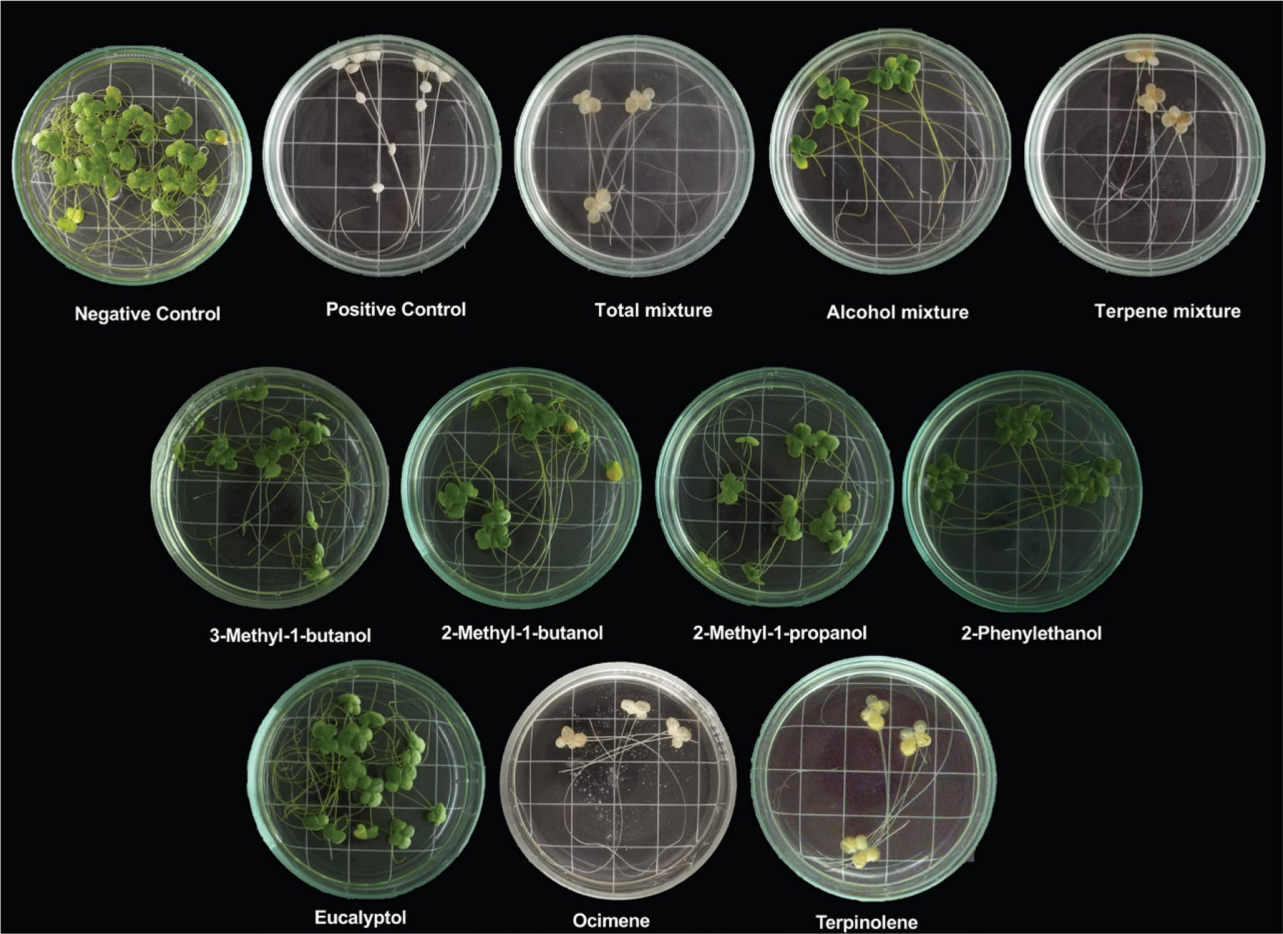
Phytotoxic effect of VOC mixtures or individual VOCs at 300 μg/mL on the aquatic plant *Lemna gibba*. *M*_*total*_ (7 VOCs; total mixture). *M*_*alcohol*_ (4 VOCs; alcohol mixture). *M*_*terpene*_ (3 VOCs; terpene mixture). Rival^®^ [glyphosate: *N-*(phosphonomethyl)glycine] was used as positive control (300 μg/mL)

A comparative evaluation of the individual VOCs or VOCs’ mixtures demonstrated a significant phytotoxic effect on *L. gibba* at 300 µg/mL (Figs. 5 and 6). The *M*_*total*_ and *M*_*terpene*_ along with the monoterpenes terpinolene and ocimene, caused complete inhibition of both frond number and dry weight of duckweed. While *M*_*alcohol*_ inhibited frond number by 70% and dry weight by 32.1%. The aliphatic alcohols and 2-phenylethanol had moderate inhibitory effects, inhibiting frond number by 32.1% to 52.5% and dry weight by 11.9% to 26.1%. In contrast, eucalyptol proved to be the least active compound, with only 14.2% inhibition in frond number. The treatments that resulted in total inhibition also induced a strong chlorosis effect (Fig. 6).

## Discussion

The bioherbicidal potential of VOCs, both as individual components and in mixtures, was evidenced by their inhibitory effect on germination, root growth, and respiration in four weed species. Root growth proved to be the most sensitive process, with *A. hypochondriacus* showing the greatest susceptibility. The VOCs mixtures exhibited greater phytotoxic activity than their individual components, suggesting synergistic interactions. Specifically, the *M*_*total*_ and *M*_*alcohol*_ demonstrated superior bioherbicidal potential by achieving complete inhibition of at least one physiological process at low concentrations (2.6 to 12.9 µg/mL).

The evaluation of the phytotoxic potential of individual VOCs demonstrated that 3-methyl-1-butanol was the most potent compound, causing complete inhibition in at least one physiological process at concentrations low (5–10 µg/mL). In contrast, 2-methyl-1-propanol and 2-phenylethanol displayed the lowest potential. Among the monoterpenes, eucalyptol was the only one with significant phytotoxicity on four plants; it inhibited root growth by over 90% and respiration by 20–70% at a high concentration tested (500 µg/mL).

Notably, the herbicidal potential of VOCs and their mixtures was also evidenced by their robust phytotoxic effect on the aquatic plant *L. gibba*. The *M*_*total*_ and *M*_*terpene*_ induced chlorosis and inhibited frond and dry weight production at 300 µg/mL. We determined that the monoterpenes terpinolene and ocimene are responsible for this effect, as their application resulted in complete inhibition of frond numbers and pronounced chlorosis. In addition, the three alcohols examined, including 2-phenylethanol, showed a significant phytotoxic effect, inhibiting frond numbers by more than 50%. This suggests that the direct impact of the VOCs on photosynthetic capacity, possibly through chlorophyll degradation, affects plant growth and survival (Gouda et al. 2016; Verdeguer et al. 2020).

Our results align with previous studies on the phytotoxic potential of VOCs, demonstrating their toxicity in mixtures or as pure compounds. A study by Nguyen et al. (2024) on 2-methyl-1-butanol and 3-methyl-1-butanol in *Oryza sativa* demonstrated that both VOCs have phytotoxic effects on germination and seedling growth. Specifically, they inhibited germination by 100% at 4 mM. In seedlings, 2-methyl-1-butanol caused a minimal reduction in shoot length (15.6%) and root growth (12%), whereas 3-methyl-1-butanol significantly inhibited root growth (56%) and secondary root growth (60%), although it increased shoot length (44.3%).

Morales-Soto et al. (2015) and Tena et al. (2021) evaluated the phytotoxic effect of 2-phenylethanol alone and in a mixture with p-cresol and 3-phenyl-1-propanol on *Lactuca sativa* and *Allium cepa*. Their study demonstrated that the VOCs mixture had a greater phytotoxic effect than the individual compounds, suggesting a synergistic interaction. For example, the mixture induced almost 100% inhibition in germination, root and cotyledon growth in both species, whereas 2-phenylethanol alone caused a reduction in germination and root growth of less than 50%.

Sidorova et al. (2022) demonstrated that the VOCs 3-methyl-1-butanol and 2-phenylethanol have a significant phytotoxic effect on *Arabidopsis thaliana*. At concentrations of 50–100 mM, 3-methyl-1-butanol caused 100% inhibition of seed germination and cotyledon emergence and a 70.9% reduction in fresh biomass. 2-phenylethanol was also highly effective, leading to 100% inhibition of germination and cotyledon emergence at 25 mM and a 72.4% reduction in fresh biomass at 200 mM. These findings highlight the strong inhibitory potential of these VOCs on plant growth.

Various studies show that VOCs from fungi have a significant phytotoxic effect on plants, affecting germination, root growth, and chlorophyll content. Muria-Gonzalez et al. (2020) found that the 3-methyl-1-butanol from *Parastagonospora nodorum* is particularly potent, completely inhibiting root growth and 83% of coleoptile growth in *Triticum aestivum* at 88.2 µg/mL. The alcohols 2-methyl-1-butanol and 2-phenylethanol exhibited a moderate inhibitory effect. 2-methyl-1-butanol inhibited root and coleoptile growth by 55% to 60%, while 2-phenylethanol inhibited them by 50% to 75%. In contrast, 2-methyl-1-propanol was less potent, with an inhibition in both processes ranging from 31% to 36%. Lee et al. (2016) confirmed that VOCs from *Aspergillus versicolor* strains, including 2-methyl-1-butanol and 3-methyl-1-butanol, reduce leaf size and number, root growth, and chlorophyll content in *A. thaliana*, with marked seedling growth inhibition at 0.5 µL/L.

Endophytic fungi have been shown to produce VOC mixtures with notable phytotoxicity. For instance, Ulloa-Benítez et al. (2016) identified that the endophytic fungus *H. anthochroum* produces 2-phenylethanol, a compound that exhibited a strong inhibitory effect on *M. sativa*, with IC₅₀ values as low as 21.4 µg/mL for root growth. Similarly, Sánchez-Ortiz et al. (2016) found that the endophytic fungus *Xylaria* sp. produces 2-methyl-1-butanol and 2-methyl-1-propanol, which both showed significant phytotoxicity. 2-methyl-1-butanol was particularly potent, with an IC₅₀ value of 4.59 µg/mL for the inhibition of *A. hypochondriacus* root growth, and 2-methyl-1-propanol also proved phytotoxic in *A. hypochondriacus* and *S. lycopersicum*, with IC₅₀ values of up to 26.45 µg/mL for root growth.

Finally, Splivallo et al. (2007) demonstrated that 3-methyl-1-butanol and 2-phenylethanol, two of the most abundant truffle VOCs, have a significant phytotoxic effect on *A. thaliana* seed germination and root growth at 30 µg/mL. 3-methyl-1-butanol was the most active, inhibiting all two processes studied, while 2-phenylethanol caused 50% bleaching in cotyledon leaves. These findings corroborate our results on the high, broad-spectrum phytotoxic activity of aliphatic alcohols like 3-methyl-1-butanol and 2-methyl-1-butanol.

Monoterpenes such as eucalyptol, ocimene, and terpinolene are abundant components of the essential oils (EOs) of various plant species. The phytotoxic potential of these compounds has been extensively studied. Among them, eucalyptol has been identified as the most potent and widely distributed monoterpene.

A recent study by Pouresmail et al. (2024) demonstrated that the EO of *Artemisia austriaca*, along with its primary monoterpenes eucalyptol and camphor, has a potent phytotoxic effect on *Avena fattua*. At 200 µg/mL, all treatments completely inhibited germination. Notably, eucalyptol at 150 µg/mL caused over 80% inhibition in plumule, root, and seedling growth, and a significant reduction in biomass. Similar findings were reported by Gouda et al. (2016), who found that eucalyptol inhibited *Echinochloa crus-galli* with IC_50_ values between 4.43 and 7.76 mM for germination, root, and seedling growth; and by Barton et al. (2014) who also who demonstrated that the *Eucalyptus* EO and its major component, eucalyptol, had a strong phytotoxic effect on post-emergence root and seedling growth in both *Raphanus sativus* and *Lolium rigidum*. At concentrations over 100 mM, eucalyptol inhibited the root and seedling growth of *R. sativus* by over 90% and 60%, respectively. *L. rigidum* seeds showed even greater sensitivity, with approximately 95% inhibition of root growth at 31.6 mM and complete inhibition of seedling growth at 100 mM. Complementary results were obtained by Li et al. (2020) found that *Lolium* sp. seed germination and root growth were inhibited by up to 100% depending on the specific EO. In addition, pure eucalyptol significantly reduced germination by approximately 20%, root growth by 25%, and seedling development by 40%. Similarly, Zhang et al. (2012) demonstrated that eucalyptol in *Eucalyptus* EOs significantly inhibited germination by around 60%, root growth by 80%, and seedling growth by 70% in *Solanum elaegnifolium.*

On the other hand, Jiang et al. (2021, 2022) investigated the phytotoxic activity of the EOs from two *Artemisia* species and their major compounds, such as eucalyptol, α-thujone, linalool, and β-myrcene. Eucalyptol had a phytotoxic effect with IC_50_ values for root growth ranging from 1,520 to 17,800 µg/mL, indicating species-specific responses. The study found synergy in the mixture of eucalyptol and α-thujone, which was more potent than the pure compounds, while the mixture of eucalyptol, linalool, and β-myrcene showed no enhanced effect. These results align with the findings of Li et al. (2020), and Zhang et al. (2012), who also demonstrated the strong phytotoxic potential of eucalyptol-rich EO in various plants.

Other monoterpenes such as terpinolene and ocimene also have considerable phytotoxic potential. For instance, Ricci et al. (2017), demonstrated that terpinolene, a component of *Monarda didyma* EO, significantly inhibited germination (57–74%) and root growth (85–94%) in *Papaver rhoeas*, *Taraxacum officinale*, and *A. fatua* at a concentration of 0.115 µg/mL.

Endophytic fungi of the genus *Hypoxylon* produce VOCs such as eucalyptol, ocimene, and terpinolene. Eucalyptol is the most abundant, with proportions of up to 60% (Macías-Rubalcava et al. 2018; Sánchez-Fernández et al. 2016; Ulloa-Benítez et al. 2016; Suwannarach et al. 2013; Tomsheck et al. 2010). A study by Ulloa-Benítez et al. (2016) reported that a VOCs mixture from *H. anthochroum* inhibited root growth of *A. hypochondriacus* with a eucalyptol IC_50_ value of 11.6 µg/mL in a gas-phase assay. These findings indicate that fungal volatiles can be as effective as plant EOs in this type of assay.

Ocimene, though less frequently reported, has also demonstrated notable phytotoxic potential. A study by Kausar et al. (2022) found that a fungal extract from *Alternaria gaisen* rich in ocimene (27.6%) significantly inhibited the germination of *Parthenium hysterophorus* seeds by 88% at a concentration of 0.1% m/v.

These findings reinforce the potent phytotoxic potential of monoterpenes, especially eucalyptol, which shows high activity across a wide range of plant species. Notably, the synergistic effects observed in VOC mixtures underscore the importance of evaluating both individual compounds and their combinations to explore their potential as bioherbicides.

Although most of the selected VOCs have been extensively investigated and their phytotoxic potential has been demonstrated in different plant species, studying their phytotoxicity mainly on the germination and growth of the root, cotyledon, hypocotyl or coleoptile, as well as the biomass. It is imperative to underscore the fact that, except for eucalyptol and 3-methyl-1-butanol (for which further details are provided below), the mechanisms of action and phytotoxicity of the alcohols and monoterpenes evaluated remain to be fully elucidated, particularly regarding mitochondrial respiration.

Our investigation demonstrates a clear correspondence between the phytotoxic effects of VOCs and their impact on basal mitochondrial respiration, a little-explored area of study. The VOC mixtures, particularly the *M*_*total*_ and *M*_*alcohol*_, showed higher phytotoxicity than pure compounds, inhibiting respiration approximately 80% at low concentration (20 µg/mL). In contrast, the *M*_*terpene*_ required concentrations 40 times higher for a similar effect. The aliphatic alcohol 3-methyl-1-butanol was the most potent individual compound, with activity comparable to *M*_*total*_ and *M*_*alcohol*_, 2-methyl-1-butanol and 2-methyl-1-propanol were progressively less active, while the aromatic alcohol 2-phenylethanol was the least active. The monoterpenes eucalyptol and ocimene showed lower activity, requiring concentrations of up to 1000 µg/mL to inhibit respiration by 60–70%. Our findings not only confirm the high phytotoxic activity of alcohols and monoterpenes but also highlight the superiority of mixtures in inducing a greater biological effect, which underscores the potential of VOCs as herbicidal agents.

In a similar manner, the mΔψ demonstrated a high degree of sensitivity to the presence of *M*_*total*_, resulting in approximately 80% damage (10 µg/mL). *M*_*alcohol*_ resulted in membrane integrity impairment that was comparable to the level of impairment caused by *M*_*total*_ at a double concentration (20 µg/mL). To a lesser extent, the mΔψ was damaged by the terpenes, with approximately 60% of the compound being affected at a concentration that was 100-fold higher than the *M*_*total*_ concentration of 1000 µg/mL. The alcohols 3-methyl-1-butanol and 2-methyl-1-butanol induced the most pronounced damage to the mΔψ, affecting approximately 90% at the highest test concentrations (20 and 30 µg/mL, respectively). 2-Methyl-1-propanol demonstrated a comparable effect at a concentration five times higher than that of 3-methyl-1-butanol (100 µg/mL). Once more, among the monoterpenes, eucalyptol and ocimene showed the strongest deleterious effects on membrane integrity, compromising approximately 90% and 70%, respectively, at the maximum test concentration (1000 µg/mL). In contrast, terpinolene inflicted a comparatively minimal impairment (38.8%).

These results strongly suggest that the data from VOC mixtures or individual VOCs are capable of disrupt cellular respiration and mitochondrial membrane potential. Mitochondrial dysfunction can alter plant energy metabolism, including a reduction in ATP synthesis, which undoubtedly alters primary metabolism, affecting growth and development, and can generate hypersensitivity to pathogens and environmental stress, embryonic lethality, pollen abortion, alterations in the biosynthesis of secondary metabolites, among other disorders, often leading to cell death (Li et al. 2024; Xu et al. 2020).

Of the VOCs studied, only eucalyptol and 3-methyl-1-butanol have evidence of effects on some fundamental physiological processes in plants. Specifically, eucalyptol has been demonstrated to inhibit DNA synthesis (Koitabashi et al. 1997), particularly in the root apical meristem (Nishida et al. 2005). It also inhibits all phases of mitosis (Romagni 2000), increases root fatty acids (Zunino and Zygadlo 2004, 2005), and inhibit chlorophyll synthesis (Gouda et al. 2016; Huang et al. 2024; Pouresmail et al. 2024). In addition, eucalyptol alters antioxidant enzyme activity and increases reactive oxygen species (ROS), malondialdehyde (MDA) content, and relative electrolyte leakage (REL) (Pouresmail et al. 2024). In a separate study, 3-methyl-1-butanol was demonstrated to cause stomatal closure in *A. thaliana* and *Nicotiana benthamiana* (Truong et al. 2024). Despite these findings, few studies have explored the underlying mechanisms arising from VOC exposure to plant energy metabolism. Early work by Muller et al. (1969) demonstrated that the eucalyptol inhibited mitochondrial respiration of *A. fatua*. Later, Abrahim et al. (2000, 2003) found that eucalyptol, limonene, and α-pinene inhibit the coupled respiration of mitochondria in *Zea mays*.

## Conclusion

Our results provide strong evidence that VOCs’ mixtures or individual VOCs exhibited high bioherbicidal potential on herbaceous and aquatic plants. For the herbaceous plants, *M*_*total*_ demonstrated considerable bioherbicide potential, while *M*_*alcohol*_ has a greater contribution to the phytotoxic effect caused by the presence of *M*_*total*_ than *M*_*terpene*_. Furthermore, 3-methyl-1-butanol has been identified as a primary contributor to phytotoxic activity in *M*_*total*_ and *M*_*alcohol*_. In general, the bioherbicide potential of VOCs’ mixtures or individual VOCs can be established as follows: *M*_*total*_>*M*_*alcohol*_>*M*_*terpene*_ >3-methyl-1-butanol >2-methyl-1-butanol >2-methyl-1-propanol >2-phenylethanol >eucalyptol >ocimene > terpinolene. The bioherbicide potential of the aquatic plant *L. gibba* can be established as follows: *M*_*total*_ = *M*_*terpene*_ = ocimene = terpinolene > *M*_*alcohol*_ >2-phenylethanol > 3-methyl-1-butanol > 2-methyl-1-butanol > 2-methyl-1-propanol > eucalyptol. The phytotoxic activity of *M*_*total*_ and *M*_*terpene*_ has been primarily attributed to the monoterpenes terpinolene and ocimene.

With respect to the mode of action, individual VOCs and their mixtures have been shown to alter respiration and mitochondrial membrane potential in intact mitochondria isolated from *M. sativa* seedlings. The VOC mixtures demonstrated a higher degree of phytotoxicity, as evidenced by alterations in respiration and mitochondrial membrane potential, in comparison to the pure compounds. Among the mixtures, *M*_*total*_ and *M*_*alcohol*_ exhibited notably more pronounced activity compared to *M*_*terpene*_. Consequently, the individual VOCs or VOCs’ mixtures examined in this study are promising candidates for the development as environmentally friendly bioherbicides in agricultural production. These VOCs have specific targets of action, including mitochondrial respiratory inhibitors and mitochondrial membrane integrity/permeability disruptors, as well as photosynthetic pigments inhibitors and shikimic acid pathway inhibitors. This will drive the advancement of bioherbicides with a dual mode of action, significantly enhancing their effectiveness in weed management.

In light of the above, and primarily based on our results, we will focus in the near future on investigating whether the VOCs used in this work are able to inhibit oxidative phosphorylation, specifically binding to some complex of the mitochondrial respiratory chain and thus blocking the flow of electron transport and inhibiting ATP synthesis. Concurrently, and contingent on the findings of chlorosis in the aquatic plant *L. gibba*, an investigation will be conducted into a secondary mechanism of action concerning the biosynthesis of photosynthetic pigments, including chlorophylls and carotenoids, as well as inhibitors of the shikimic acid pathway.

## Data Availability

All data generated or analyzed during the present study are included in this published article.
